# Dipeptidyl peptidase-4 inhibitors, pancreatic cancer and acute pancreatitis: A meta-analysis with trial sequential analysis

**DOI:** 10.1038/s41598-017-19055-6

**Published:** 2018-01-15

**Authors:** Lana C. Pinto, Dimitris V. Rados, Sabrina S. Barkan, Cristiane B. Leitão, Jorge L. Gross

**Affiliations:** Division of Endocrinology, Hospital de Clínicas de Porto Alegre/Universidade Federal do Rio Grande do Sul, Ramiro Barcelos St, 2350, Prédio 12, 4th floor, ZIP 90035-903 Porto Alegre, Brazil

## Abstract

The use of dipeptidyl peptidase-4 (DPP-4) inhibitors may be associated with pancreatic cancer and acute pancreatitis. Recent meta-analyses have reported conflicting findings. Therefore, we performed a meta-analysis to assess the risk of both pancreatic cancer and acute pancreatitis associated with the use of DPP-4 inhibitors. We also used trial sequential analysis to evaluate whether the number of patients included was enough to reach conclusions. We included randomised controlled trials lasting 24 weeks or more that compared DPP-4 inhibitors with placebo or other antihyperglycaemic agents. A total of 59,404 patients were included. There was no relationship between the use of DPP-4 inhibitors and pancreatic cancer (Peto odds ratio 0.65; 95% CI 0.35–1.21), and the optimal sample size was reached to determine a number needed to harm (NNH) of 1000 patients. DPP-4 inhibitors were associated with increased risk for acute pancreatitis (Peto odds ratio 1.72; 95% CI 1.18–2.53), with an NNH of 1066 patients, but the optimal sample size for this outcome was not reached. In conclusion, there is no association between DPP-4 inhibitors and pancreatic cancer, and a small risk for acute pancreatitis was observed with DPP-4 inhibitor use, although the latter finding is not definitive.

## Introduction

Dipeptidyl peptidase-4 (DPP-4) inhibitors, or gliptins, are incretin-mimetic oral antihyperglycaemic agents whose clinical use has steadily increased over the past ten years^1^. These medications are not associated with severe hypoglycaemia and have a neutral effect on weight. However, there are concerns that the use of DPP-4 inhibitors may be associated with increased risk for pancreatic cancer and acute pancreatitis^2,3^.

An early study analysed the FDA reports of pancreatic cancer and concluded that there was a 2.7-fold increase in the risk for pancreatic cancer with DPP-4 inhibitor use^2^. Another study suggested that DPP-4 inhibitor use was associated with the occurrence of α-cell hyperplasia, that is, increased proliferation and dysplasia, with potential evolution into neuroendocrine tumours^4^. Later, a pooled analysis of clinical trials with sitagliptin suggested no association between use of this medication and pancreatic cancer^5^. The lack of association between DPP-4 inhibitor use and pancreatic cancer was evaluated in a pooled analysis including only two large randomised trials, and no association was found^6^. Recently, three meta-analyses assessed the risk for acute pancreatitis among patients taking gliptins. Li *et al*. analysed the results of 60 randomised and non-randomised trials and found no increased risk of pancreatitis in patients treated with gliptins compared with controls^7^. Despite this reassuring finding, the inclusion of observational studies might have influenced the results owing to selection bias. Conversely, two other meta-analyses found contradictory results when analysing the results of three large studies assessing the cardiovascular risk of sitagliptin, saxagliptin and alogliptin^1,3^. In these studies, the use of DPP-4 inhibitors increased the risk of pancreatitis. Importantly, the potential cases of acute pancreatitis were adjudicated in these three trials.

Considering the potential association between DPP-4 inhibitor use and both pancreatic cancer and acute pancreatitis, we performed a meta-analysis including all randomised trials with DPP-4 inhibitor use lasting at least 24 weeks, in order to analyse whether there is an increased risk of pancreatic cancer and/or acute pancreatitis. We also applied trial sequential analysis (TSA) to assess whether the number of patients randomised in these trials was sufficient to reach definitive conclusions.

## Methods

### Protocol and registration

This systematic review and meta-analysis follows the recommendations of the Preferred Reporting Items for Systematic Reviews and Meta-analyses (PRISMA) protocol^8^ and was registered in the International Prospective Register of Systematic Reviews (PROSPERO) under the number CRD42016953346.

### Patient Involvement

No patients were directly involved in the study.

### Information source and search strategy

We performed a systematic literature search for all randomised clinical trials (RCTs) that compared DPP-4 inhibitor use with either placebo or other antihyperglycaemic medications. We searched MEDLINE, Embase, the Cochrane Central Register of Controlled Trials (CENTRAL) and ClinicalTrials.gov from database inception to May 2016. We also searched abstracts from the most recent meetings of the American Diabetes Association and the European Association for the Study of Diabetes. The search strategy used the Medical Subject Headings (MeSH) terms “sitagliptin” OR “saxagliptin” OR “linagliptin” OR “alogliptin” OR “vildagliptin” AND “diabetes mellitus, type 2” AND a validated filter to identify RCTs^9^. All eligible trials were considered for review, regardless of language. A manual search of the reference lists of key articles was also performed.

### Eligibility criteria

The inclusion criteria were: (1) RCTs, (2) DPP-4 inhibitor use versus any standard of comparison, (3) treatment for at least 24 weeks, (4) definition of events of acute pancreatitis and/or pancreatic cancer, (5) inclusion of patients ≥18 y old, and (6) diagnosis of type 2 diabetes according to the American Diabetes Association criteria^10^.

### Study selection and data collection

Two independent investigators (L.C.P. and S.S.B.) selected studies on the basis of titles and abstracts. Studies satisfying the inclusion criteria and those with abstracts that lacked crucial information to decide upon their exclusion were retrieved for full-text evaluation. Both investigators also analysed the selected trials and extracted data; disagreements were resolved by a third reviewer (D.V.R.). The following information was extracted: first author’s name, year of publication, sample size and dropouts, age distribution, gender distribution, trial duration, treatment in use prior to randomisation, acute pancreatitis and pancreatic cancer events.

### Risk of bias in individual studies and the quality of meta-analysis

The quality of studies was assessed according to the Cochrane Collaboration tool for risk of bias, including the following six domains: random sequence generation, allocation concealment, blinding, incomplete outcome data, selective reporting, and other biases such as adjudication of events^11,12^. In adjudicated trails, the diagnosis was confirmed by the following criteria: symptoms of abdominal pain or vomiting and evidence of pancreatic inflammation (e.g., elevated pancreatic enzymes, amylase or lipase >3× the normal upper limit; in patients with chronic pancreatitis, enzyme elevations >2× the normal upper limit) or evidence of acute pancreatitis documented by abdominal computerised tomography, magnetic resonance imaging or ultrasound showing focal, diffuse and inhomogeneous gland enlargement. The quality of each outcome (pancreatic cancer and acute pancreatitis) was evaluated by the Grading of Recommendations Assessment, Development and Evaluation (GRADE) approach^13^. Each meta-analysis was rated as high, moderate, low or very low quality.

### Synthesis of results

We compared the events of interest between patients randomised to the use of DPP-4 inhibitors and patients randomised to the control treatment (placebo or other antihyperglycaemic medications). The outcomes of interest were pancreatic cancer and acute pancreatitis.

Data were summarised with direct meta-analysis to compare DPP-4 inhibitors with placebo and other antihyperglycaemic agents. We used the Peto odds ratio and the Mantel-Haenszel test for analysis. We used the Peto odds ratio in the primary analyses as it is more conservative (can identify smaller associations) and is superior when dealing with rare events. Heterogeneity was assessed by Cochran’s Q test (a *p*-value of 0.1 was considered statistically significant) and the *I*^2^ test (values greater than 50% were considered to indicate elevated statistical heterogeneity). For studies with no events in either arm, continuity correction was performed to include these data in TSA analyses. To assess whether the length of the trials was related to the outcome, we performed meta-regression, using study duration as a covariate.

Furthermore, to address whether current information is sufficient for firm conclusions, we performed TSA of the identified studies. This analysis is analogous to sample size estimation or interim analysis of a single study^14,15^ and is associated with a cumulative meta-analysis represented by the Z-curve. Therefore, we calculated the sample size required to detect or reject a minimal relevant difference between DPP-4 inhibitors and the control^1,4^. We defined this minimal relevant difference as an absolute difference of 0.1% in the incidence of both outcomes (pancreatic cancer and acute pancreatitis) between groups on the basis of previous trial results^1^. We conducted the TSA with an overall 5% risk of type I error and 20% risk of type II error (power of 80%).

We evaluated publication bias with visual inspection of funnel plots and with Begg’s and Egger’s tests. If small-study bias was identified, we applied the trim-and-fill method to explore the effect of missing studies on the outcomes.

The analyses were performed using the programs RevMan version 5.3 (Cochrane Collaboration, Copenhagen, Denmark) and STATA 12.0 (Stata Inc., College Station, Texas, USA). The TSA was performed with TSA software (Centre for Clinical Intervention Research Department, Copenhagen, Denmark).

## Results

**Figure 1:**
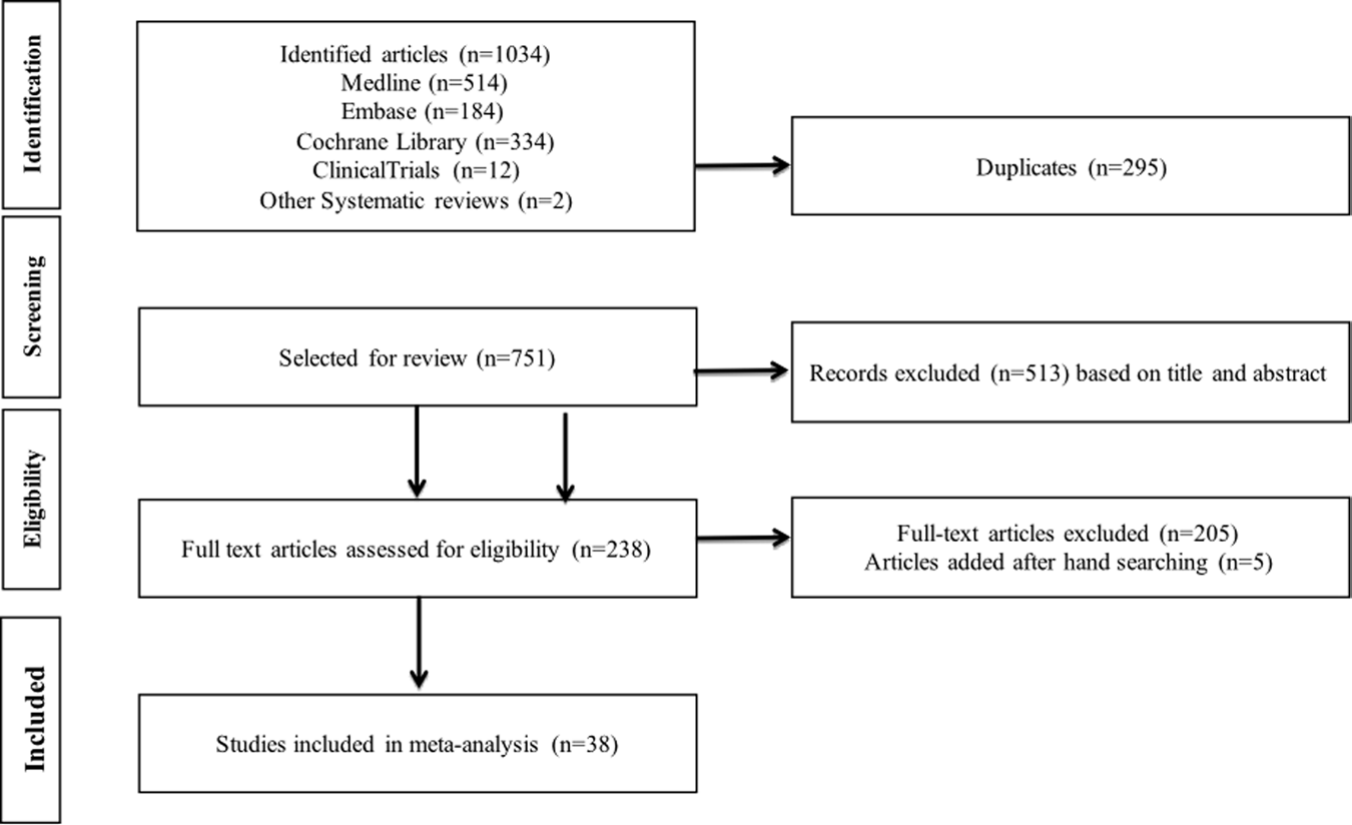
Study flowchart.

Our search retrieved 763 articles. After we scanned through the titles and abstracts and removed all duplicates, 186 articles remained for full-text evaluation. Ultimately, 38 trials were included in the analysis (Fig. 1 – Study Flowchart).

The selected studies were published between 2009 and 2015. The mean trial duration was 63.5 weeks (range, 24–260). The analysis included 59,404 patients; 39,970 (62.1%) were men, and the mean age was 57.39 ± 5.12 years. The main characteristics of the included trials are presented in Table 1. Results regarding the individual quality of the included trials are presented in Supplemental Material.

**Table 1:** Characteristics of the included trials.

Author Year	*n*	Follow-up (weeks)	Men (%)	Mean age (y)	Background treatment
Ahren 2014	1012	104	47.6	54.4	Metformin
Araki 2013	561	26	70.4	60.0	Naïve or OADs
Arechevaleta 2011	1035	30	54.4	56.3	Metformin
Arjona-Ferreira 2013	426	54	59.8	64.2	Diet, Exercise or OAD
Bajaj 2014	272	24	48.5	53.8	Metformin + Pioglitazone
Barnett 2012	455	52	41.3	58.0	Insulin or Insulin + Metformin
Bergenstal 2010	514	26	51.7	52.5	Metformin
TECOS 2015	14671	260	70.7	66.0	Metformin, Pioglitazone, Sulfonylurea or Insulin
DeFronzo 2015	674	24	53.7	56.2	Metformin
DeFronzo 2012	743	26	46.4	54.1	Metformin
Del Prato 2014	2639	104	49.7	55.4	Metformin
Fredrich 2012	366	24	45.9	54.9	Naïve
Gallwitz 2012	1552	104	60.2	59.8	Metformin
Henry 2014	1615	54	56.5	NR	Diet, Exercise, Metformin or Sulfonylurea
Hollander 2009	565	24	49.6	54.0	Thiazolidinedione
Inagaki 2013	574	52	69.9	60.9	OADs
Jadzinsky 2009	1309	24	49.2	52.0	Naïve
SAVOR-TIMI 53 2013	16492	140	66.9	65.0	Non-incretin therapies
Leiter 2014	507	52	53.7	63.3	OADs
Lewin 2015	667	24	53.8	54.6	Naïve
Mintz 2014	858	104	51.7	57.6	Metformin
Nauck 2007	1172	52	59.2	56.7	Metformin
Nauck 2014	1098	104	46.5	54.1	Metformin
Nowicki 2011	170	52	42.9	66.5	OADs or Insulin
Olansky 2011	1250	44	56.8	49.7	Diet + Exercise
Pfutzner 2011	1306	76	49.2	52.0	Naïve
Pratley 2012	665	52	52.9	55.3	Metformin
Rosenstock 2009	401	24	50.9	53.5	Naïve
Rosenstock 2009	390	26	41.3	NR	Insulin
Rosenstock 2010	655	26	48.9	52.6	Naïve
Schernthaner 2013	756	52	55.9	56.7	Metformin + Sulfonylurea
Schernthaner 2015	720	52	61.8	72.6	Metformin
Seck 2010	1172	104	59.2	56.7	Metformin
Sheu 2015	1261	52	52.2	60.0	Insulin
Wainstein 2012	521	32	53.6	52.3	Diet + Exercise
EXAMINE 2013	5380	208	67.9	60.9	OADs
Weistock 2015	1098	26	47.4	54	Metformin
Williams-Herman 2012	306	24	52.0	53.7	Diet + Exercise

OADs, oral antidiabetics; NR, not reported.

The analysis of the funnel plots and Begg’s and Egger’s tests suggested no publication bias for either outcome (pancreatic cancer or acute pancreatitis).

### DPP-4 inhibitors and pancreatic cancer

**Figure 2:**
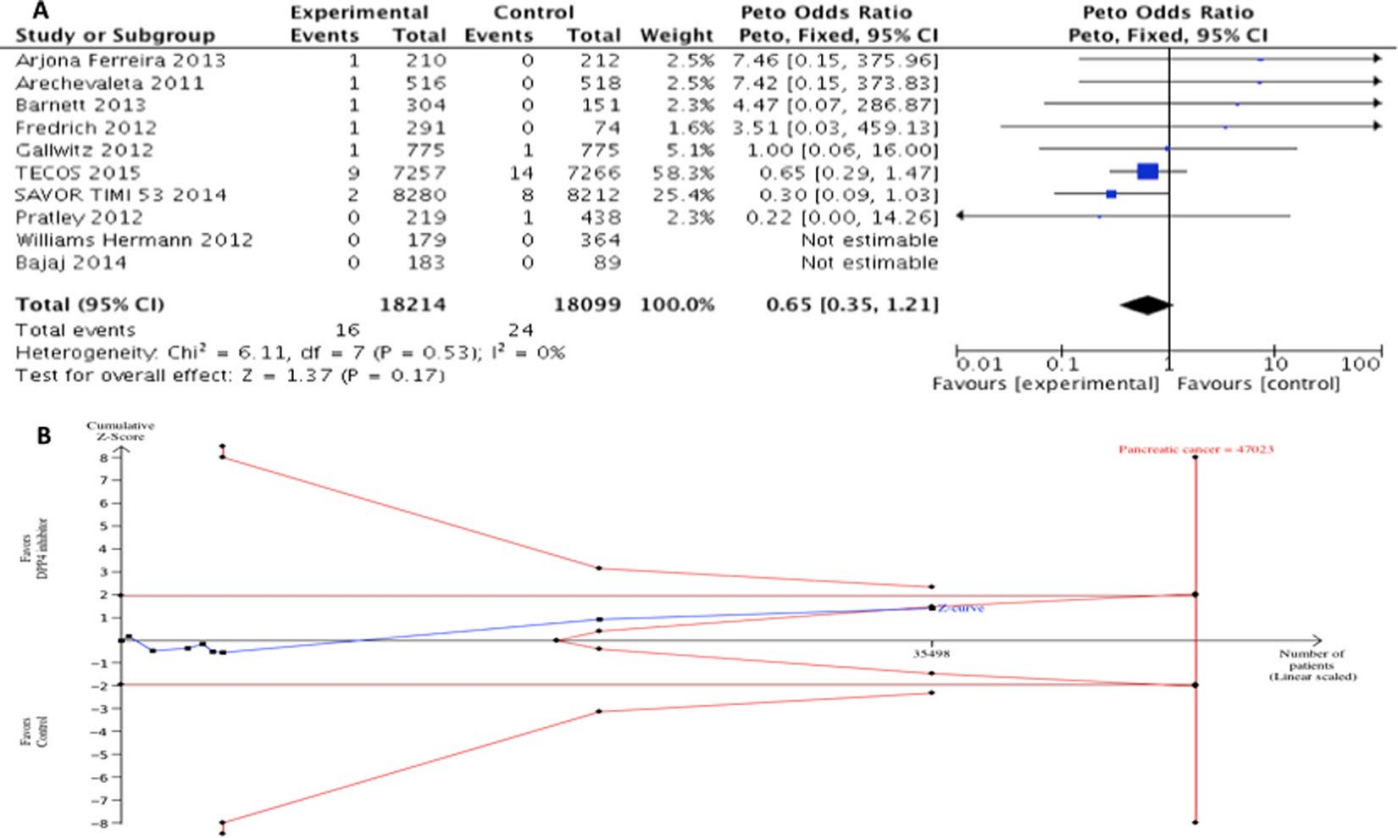
(**A**) Forest plot for association between DPP-4 inhibitors and pancreatic cancer; (**B**) TSA for pancreatic cancer.

There were 16 events of pancreatic cancer in the DPP-4 inhibitor group and 24 events in the control group. DPP-4 inhibitors were not associated with increased risk for pancreatic cancer in the direct meta-analysis (Peto odds ratio 0.65; 95% CI 0.35–1.21) (Fig. 2A **–** Forest plot for association between DPP-4 inhibitors and pancreatic cancer). Similar results were observed with the Mantel-Haenszel test (0.65; 95% CI 0.35–1.19). When we performed TSA, DPP-4 inhibitors were still not associated with pancreatic cancer (Peto odds ratio 0.66; 95% CI 0.36–1.19), and the number of randomised patients for this outcome surpassed the futility boundary (Fig. 2B – TSA for pancreatic cancer). Meta-regression did not show an interference of study duration with the outcome (p = 0.867; 8 studies included) (Supplemental Material).

### DPP-4 inhibitors and acute pancreatitis

There were 64 events of acute pancreatitis in the DPP-4 inhibitor group and 39 events in the control group. DPP-4 inhibitors were associated with an increased risk of acute pancreatitis in direct meta-analysis (Peto odds ratio 1.72; 95% CI 1.18–2.53; Supplemental Material) and with an absolute risk difference of 0.1% (representing a number needed to harm (NNH) of 1066). Mantel-Haenszel analysis showed comparable results (1.52; 95% CI 1.05–2.18). As we aimed to be conservative, TSA was performed to assess whether there was enough information to reach a definite conclusion regarding the association between DPP-4 inhibitors and acute pancreatitis. For this outcome, the number of patients evaluated (n = 59,404) did not reach the optimal sample size (n = 140,665), and the boundaries of benefit, harm and futility were not crossed (Peto odds ratio 1.34; 95% CI 1.00–1.79). In meta-regression, no interference of study duration with acute pancreatitis was seen (p = 0.252; 25 studies included).

## Discussion

The results of the present review indicate that the use of DPP-4 inhibitors is not associated with increased risk for pancreatic cancer. Furthermore, the TSA meta-analysis confirmed that the number of patients available was enough to reach this conclusion. There seemed to be an association between the use of DPP-4 inhibitors and acute pancreatitis, although the number of randomised patients was not sufficient for a firm conclusion and the estimated risk of acute pancreatitis is small (one patient in 1066 patients treated with DPP-4 inhibitors).

Concern regarding the association between DPP-4 inhibitor use and pancreatic cancer was raised after a review of cases reported by the FDA^2^. Other studies have suggested an association between DPP-4 inhibitor use and pancreatic cancer^4,5^, but there is still an ongoing debate on this topic. Additionally, several observational studies have explored the association between DPP-4 inhibitors and pancreatitis^16,17^. However, owing to study design characteristics, the results may be affected by selection and confounding biases. As there are a great number of randomised trials evaluating these medications, a systematic review and meta-analysis of these studies is recommended to properly address this clinical question.

Before this review, three other meta-analyses evaluated the association between clinical use of DPP-4 inhibitors and acute pancreatitis. The first one^7^ did not find an association between use of DPP-4 inhibitors and acute pancreatitis; however, this review included not only randomised trials but also prospective and retrospective observational cohort studies. Most importantly, the events were not adjudicated. The other two^1,3^ found an increased risk of acute pancreatitis in patients treated with DPP-4 inhibitors; however, they included only three large cardiovascular randomised trials, namely, EXAMINE, SAVOR-TIMI 53 and TECOS^18–20^. In these trials, a specialised committee adjudicated the diagnosis of acute pancreatitis. None of these reviews performed TSA to evaluate whether the results were definitive, and, more importantly, none of them evaluated the risk of pancreatic cancer associated with use of DPP-4 inhibitors.

Our study adds new information regarding this point. It included all randomised trials with DPP-4 inhibitor use that lasted for at least 24 weeks and, through TSA meta-analysis, evaluated whether the number of cases was sufficient to support the conclusions. There was a small risk of acute pancreatitis, such that it would be necessary to treat 1066 patients to have one case of acute pancreatitis, but the number of patients included in the meta-analysis was not sufficient to support this conclusion. Notably, owing to the large number of diabetic patients using DPP-4 inhibitors worldwide, a great number of cases of acute pancreatitis might be prevented by taking into account pre-existing risk factors for acute pancreatitis, such as gallstones and hypertriglyceridaemia, when considering whether to prescribe this type of medication.

On the other hand, GLP-1 agonist use is not associated with an elevated risk of acute pancreatitis, as recently revealed by a meta-analysis from Storgaard *et al*.^21^. Receptors for GLP-1 are largely found in the pancreatic ducts and the pancreatic islets. Acinar and duct cells respond to GLP-1 therapy with proliferation^22,23^. A previous study in rats exposed to sitagliptin reported haemorrhagic pancreatitis in one rat and acinar-to-ductal metaplasia in others^24^. Therefore, the association between incretins and acute pancreatitis is biologically plausible. However, it remains unclear why DPP-4 inhibitors are associated with pancreatitis and GLP-1 agonists are not^21^.

Regarding pancreatic cancer, no association between use of gliptins and pancreatic cancer was observed, and TSA meta-analysis showed that there were enough patients randomised for this observation.

The main limitation of our meta-analysis was the duration of the trials (mean of 63.5, minimum and maximum of 24 and 260 weeks), which may be insufficient to evaluate the development of pancreatic cancer. We tried to overcome this limitation by including study duration as a covariate in the meta-regression, and this variable did not have an influence on the outcome. However, we must consider that this analysis might have low power owing to the number of included trials. Another limitation is the criteria used for diagnosis of acute pancreatitis in trials. In adjudicated trails, the diagnosis was confirmed by an adjudication committee and the criteria used were clearly described. However, in non-adjudicated trials, the criteria used are less straightforward. Nonetheless, restricting the analysis to adjudicated studies did not change the results. Furthermore, owing to the design of the present study, we were not able to explore whether there is a specific subgroup of diabetic patients with increased susceptibility to acute pancreatitis. The included trials did not describe the risk factors for this complication, such as hypertriglyceridaemia, alcohol consumption, and previous history of cholelithiasis. The only factor classically associated with acute pancreatitis that was mentioned was smoking status, which was similar in the intervention and control groups.

Finally, there is enough information to suggest a lack of association between the use of DPP-4 inhibitors and pancreatic cancer, but not acute pancreatitis. The latter seems to be a continued concern, and additional study data are needed. Despite this uncertainty, the apparent risk is small.

## Electronic supplementary material

Supplementary Dataset 1

Supplementary Dataset 2
