# Supplementary material for: Dipeptidyl peptidase-4 inhibitors, pancreatic cancer and acute pancreatitis: A meta-analysis with trial sequential analysis
Source: Sci Rep. 2018 Jan 15;8:782. doi: 10.1038/s41598-017-19055-6 (PMC5768864; doi:10.1038/s41598-017-19055-6)
Supplement: Supplementary file 1 — Supplementary Dataset 1 [file 41598_2017_19055_MOESM1_ESM.doc]

**Dipeptidyl Peptidase-4 inhibitors, pancreatic cancer and acute pancreatitis: A meta-analysis with trial sequential analysis**

Running title: Acute pancreatitis and pancreatic cancer with DPP-4 inhibitor use

Lana C. Pinto M.D., Dimitris V. Rados M.D., Sabrina S. Barkan M.D., Cristiane B. Leitão M.D., Jorge L. Gross M.D.

**Supplemental Material**


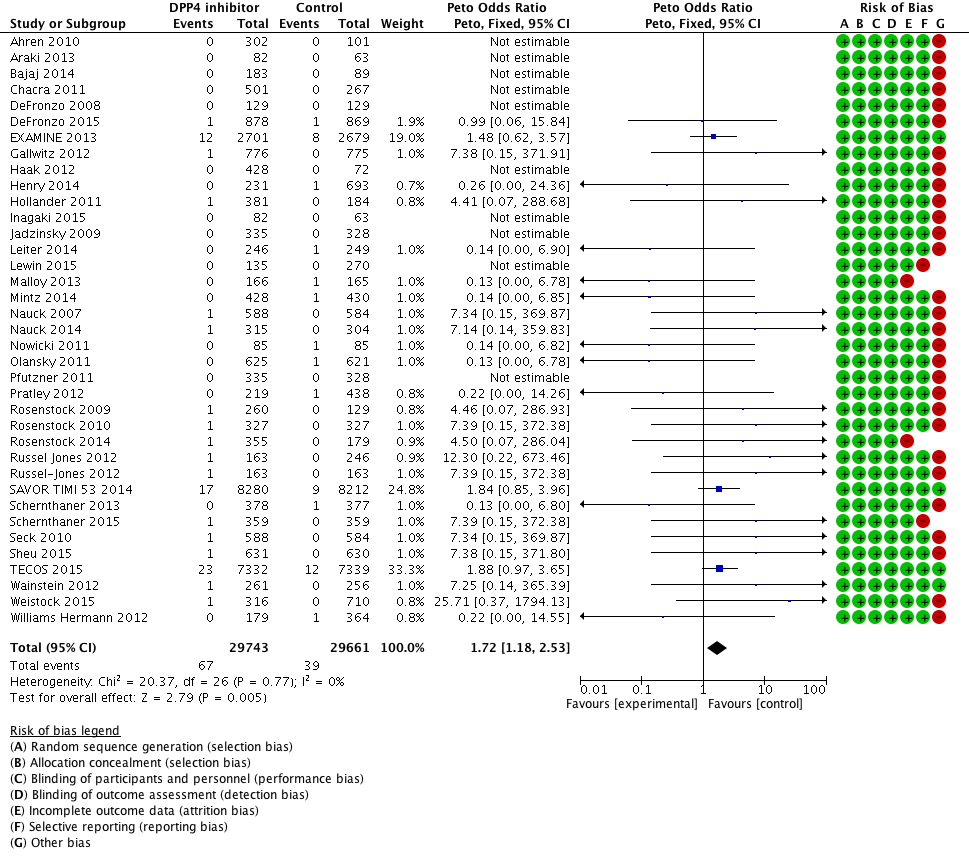


**1S.** Forest Plot for DPP-4 inhibitors on acute pancreatitis and risk of bias in included trials

Number of studies =8

Coef. -0.0006

95% CI -0.009 to 0.008

Length of Study (weeks)

**2S**. Meta-regression for pancreatic cancer using length of study as covariate

Number of studies = 25

Coef. 0.005

95% CI -0.004 to 0.157

Length of Study (weeks)

**3S**. Meta-regression for acute pancreatitis using length of study as covariate
